# Consistent Value of Two-Stage Pedicle Flaps in the Age of Microsurgical Maxillofacial Reconstruction

**DOI:** 10.1007/s12663-021-01635-9

**Published:** 2021-08-24

**Authors:** G. Frohwitter, R. Lutz, C. Baran, M. Weber, C. P. Nobis, A. Rau, M. Kesting

**Affiliations:** 1 Department for Oral and Maxillofacial Surgery, University Hospital Erlangen, Glueckstrasse 11, 91054 Erlangen, Germany; 2 Department for Oral and Maxillofacial Surgery, University Hospital Greifswald, Greifswald, Germany

**Keywords:** Pedicle flaps, Reconstructive surgery, Back-up procedures, Transplant autonomy

## Abstract

**Introduction:**

Up to the second half of the twentieth century, pedicled flaps marked the gold standard in reconstructive surgery. Followed by the introduction of microsurgical techniques, these flaps were increasingly abandoned. We conducted a retrospective study to determine the value of two-stage pedicle flaps in modern maxillofacial reconstruction.

**Material & Methods:**

A chart review from October 2017 to November 2020 was performed to identify patients who were treated by a two-stage pedicle flap in our Department of Oral and Maxillofacial Surgery.

**Results:**

A total of 31 patients, 17 female and 14 males received 36 two-stage pedicle flaps. All patients were in noticeably impaired health condition with a majority of ASA-score 3. The defect location mainly contained extraoral resections (58.3%). A variety of flaps were harvested consisting of buccal flaps, Abbe flaps, forehead flaps, deltopectoral flaps, nasolabial flaps, and a tubed flap.

**Discussion:**

The study outlines two indications for the use of two-stage pedicle flaps. Firstly, as a back-up strategy in heavily pre-treated wound beds and secondly in an almost contrarily indication as a first-choice reconstructive option of the facial skin in esthetic demanding cases.

**Conclusion:**

The timesaving and straight forward surgical approach as well as their low postsurgical complications and strong long-time success rates secure the two-stage pedicle flap a justified niche role in times of microsurgical maxillofacial reconstruction.

**Supplementary Information:**

The online version contains supplementary material available at 10.1007/s12663-021-01635-9.

## Introduction

Maxillofacial reconstruction is performed for the functional and esthetic rehabilitation of a patient and may be essential for speech, ingestion, and emotional interaction. A key algorithm of successful reconstruction is the coalition of all the basic principles of wound closure [1]. Pedicle flaps are no longer the key procedure in reconstructive surgery since the evolution of maxillofacial reconstruction from use of primary sutures to microvascular surgery. The history of regional pedicle flaps dates back to 500 BC, when a nasal reconstruction was performed with a forehead flap [2]. Up to the second half of the twentieth century, pedicle flaps marked the gold standard in reconstructive surgery. However, after the introduction of microsurgical techniques in the 1980s, pedicle flaps have been increasingly abandoned. The considerably high success rates of > 95% in microsurgical centers, versatile fields of application, and possibility of reconstructing extensive defects are some advantages of microsurgery [3]. However, owing to the maintenance of blood supply, straightforward method of harvest, and favorable esthetic outcomes in terms of skin color and tissue texture, pedicle flaps have secured a niche role in reconstructive surgery.

The primary indications of a pedicle regional flap are highly contrasting, as they are the last option for defect closure of hostile wound beds and the first choice in esthetic reconstruction of facial units after skin tumor removal or cleft surgery. In case of extensive defects, the patient’s health status is the prime reason to avoid microvascular reconstruction and opt for a local pedicle flap because of the significantly reduced operation time [4]. Furthermore, the constitution of the wound bed is essential, as irradiated and infectious tissues are a risk factor for consecutive flap failure. If microvascular approaches in vessel-depleted necks or after flap loss appear unfavorable, extensive flaps such as the deltopectoral flap should be considered, which constitute a straightforward and reliable surgical approach to minimize patient morbidity.

Smaller pedicle flaps such as the paramedian forehead flap or the Abbe flap that are intended for extraoral reconstruction are the first choice in advanced nasal or lip reconstruction. The tissue characteristics in terms of color and texture are esthetically ideal for local skin or vermilion reconstruction. Nevertheless, two-stage pedicle flaps have the disadvantage of a temporary but significant facial deformity due to the visible tissue bridge, which is essential for sufficient blood supply and complete healing. As patients often consider this condition a severe limitation in terms of social interaction and body image, long-term advantages are not apparent initially. Therefore, patient education is essential for successful reconstruction.

The following article describes the indications of the two-stage pedicle flaps in modern maxillofacial reconstruction and discusses the unaddressed issue of the ideal time for pedicle dissection.

## Material & Methods

This retrospective review was performed in accordance with the Declaration of HELSINKI and approved by the local ethics committee, and all patients provided written informed consent for publication.

Statistical analysis was performed using SPSS 24 (Released 2016. IBM SPSS Statistics for Windows, IBM Corp., Armonk, NY).

### Patients and Clinical Data

A retrospective medical record review was performed from October 2017 to November 2020 to identify patients who were treated using two-stage pedicle flaps at the Department of Oral and Maxillofacial Surgery (main inclusion criteria). Data were retrieved from the electronic database of the institution. The demographic profile, indication for the pedicle flap, histopathological diagnosis, previous therapies, current treatment, and mode of reconstruction were recorded. Additionally, the medical records were assessed for postoperative complications such as bone exposure, flap loss, or infections. The time of pedicle dissection was recorded from the date of surgery to the date when the surgical pedicle was dissected.

## Results

### Demographic Data and Health Status

A total of 31 patients, 17 (54.8%) female and 14 (45.2%) male received 36 two-stage pedicle flaps. The mean age at the time of surgery was 58.59 years (median, 63.97 years; minimum, 12.38 years; maximum, 91.96 years). All patients had at least one secondary diagnosis (minimum 1, maximum 10, median 3.5, mean 4.33) and noticeably impaired general health, with 2 (6.5%) patients exhibiting an American Society of Anesthesiologist’s-Physical status (ASA-PS) score of 1, 9 (29.0%) with an ASA-PS score of 2, and 20 (64.5%) with an ASA-PS score of 3. Furthermore, 19 (61.3%) patients had received oncological pre-treatment other than surgery; 12 (38.7%) had received radiotherapy, 1 (3.2%) had received chemotherapy, and 6 (19.4%) had received chemoradiation.

### Underlying Disease, Type of Flap, and Defect Location

**Fig. 1:**
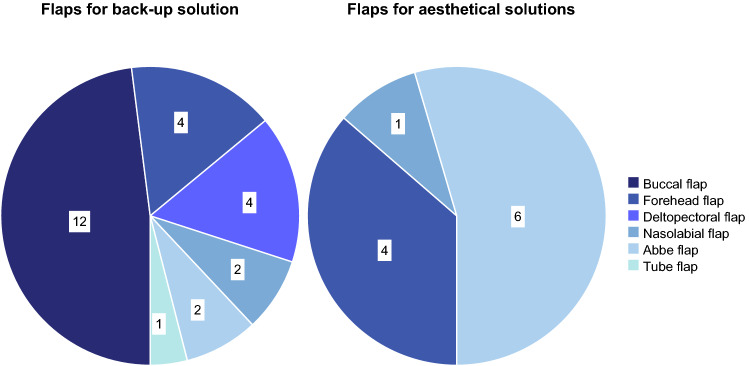
Number of pedicled flaps (*n* = 36) harvested as a back-up solution and as a primary esthetical solution

**Fig. 2:**
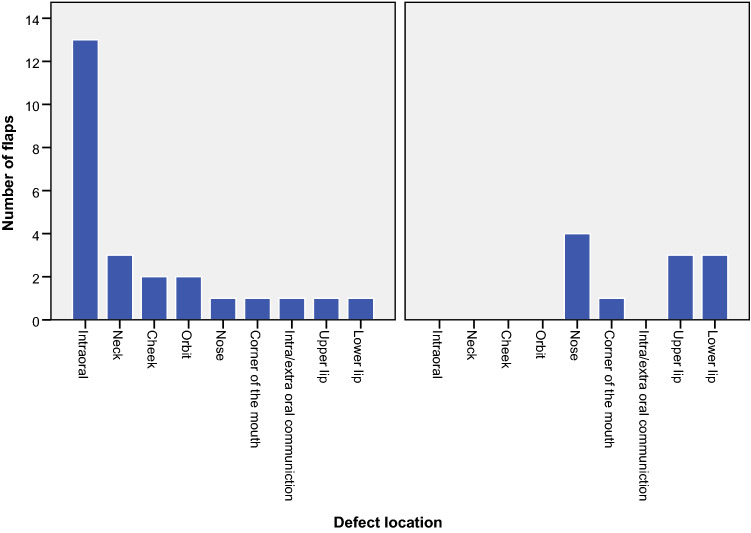
Defect locations in all patients divided in back-up situations and as a primary esthetical situation

Flap surgery was necessary in 20 (55.5%) patients with malignant tumors, 5 (13.9%) with cleft-associated conditions, 3 (8.3%) with osteomyelitis/osteoradionecrosis, 3 (8.3%) with vestibuloplasties, 2 (5.6%) with oronasal fistulas after partial microvascular flap necrosis, 1 (2.8%) with exposed bone after transpalatal distraction, 1 (2.8%) with back-up pedicle flap, and in 1 (2.8%) patient with mucosal graft after complex dental trauma and bone augmentation. In total, 25 (69.4%) of 36 flaps were harvested as back-up in heavily pre-treated wound beds, whereas 11 (30.6%) flaps were harvested as the best esthetic option for defect closure (Figs. 1 and 2).

The flaps consisted of 12 (33.4%) buccal flaps, 8 (22.2%) Abbe flaps, 8 (22.2%) forehead flaps, 4 (11.1%) deltopectoral flaps, 3 (8.3%) nasolabial flaps, and 1 (2.8%) tubed flap (Fig. 1).

The defect location consisted of 21 (58.3%) extraoral resections, 1 (2.8%) corresponding intra-extraoral fistula, and 14 (38.9%) intraoral wound beds (Fig. 2).

### Surgery Duration, Wound Healing, and Pedicle Dissection

The duration of surgery ranged from 47 to 743 min (median, 92.5 min; mean, 130.5 min). In the case that took 743 min, the initially planned microvascular flap was dismissed owing to malperfusion, and a local pedicle flap was harvested. The time difference between reflection of the back-up flap and the pedicle flap as the most esthetic option for reconstruction did not differ significantly (*p* < 0.005).

Healing was uneventful in 26 flaps (72.2%). However, the following complications occurred in the remaining flaps: 5 (13.8%) wound dehiscences, 1 (2.8%) venous congestion, 1 (2.8%) intraoral fistula, 1 (2.8%) postoperative bleeding, 1 (2.8%) intraoral wound dehiscence with an extraoral fistula, and 1 (2.8%) partial transplant loss. In 4 (11.1%) flaps, surgical reintervention was needed, of which one flap (buccal flap) could not be preserved, and a second buccal flap was needed to cover the defect completely.

All pedicles were dissected in the second surgery, which was performed under general anesthesia in 30 cases and local anesthesia in 6 cases. The duration between flap surgery and pedicle dissection was 14–106 days (mean, 33.25 days; median, 27 days). Delayed flap dissection was a result of prolonged orthodontic treatment in a patient with an oronasal fistula and a cleft palate. Except one patient (3.2%), all other patients (*n* = 30) who were treated using a two-stage pedicle flap (*n* = 36) exhibited permanent wound closure. The remaining patient, who revealed the presence of an intraoral fistula, was a 72-year-old man who had a secondary cancer of the mandible and who had been heavily pre-treated for tonsil carcinoma (surgery and chemoradiation). Thereafter, the patient developed a vessel-depleted neck with a hostile, chronically infected wound bed. Furthermore, 3 (9.7%) patients died due to local tumor recurrence within three years of aftercare.

## Discussion

In the age of microsurgery, regional pedicle flaps have retained an indispensable role in maxillofacial reconstruction. Two-stage pedicle flaps are primarily indicated in patients with severe comorbidities who require short operation times, salvage surgeries, or palliative treatment [4, 5]. The disadvantages of two-stage pedicle flaps are unfavorable esthetic appearance and need for a second surgery for pedicle dissection.

The results of this study indicate that impaired general health status favors non-microvascular solutions to shorten the operating time. The mean duration of surgery was 130.5 min, which is significantly lesser than that required for any microvascular procedure performed in the maxillofacial region [4, 6]. Moreover, grading the patient’s comorbidities and classifying the physical resilience before surgery revealed that the majority of patients (64.5%) were classified as ASA-PS score 3, which indicates substantial functional limitations and severe systemic disturbances caused by the condition to be treated by surgical intervention or by other pre-existing pathological condition [7, 8]. Furthermore, almost one-thirds of the patients (29.0%) were classified as ASA-PS score 2, indicating that the overall patient collective that primarily underwent a pedicle flap procedure showed a complex medical history of general and treatment-associated illnesses. Interestingly, we did not find any significant difference in the ASA-PS scores between patients who had undergone primary two-stage pedicle flap reconstructions for esthetic reasons and those who had undergone a back-up flap procedure (overall 2 [6.5%] patients with ASA-PS score 1; 9 [29.0%] patients with ASA-PS score 2; and 20 [64.5%] patients with ASA-PS score 3). This might be explained by the fact that most patients treated in a university hospital exhibit severe general health impairment, and the main indications to perform a two-stage pedicle flap surgery are often illnesses of an aging society such as cancer or osteo(radio)necrosis or congenital deformities, which lead to higher ASA-PS scores.

**Fig. 3:**
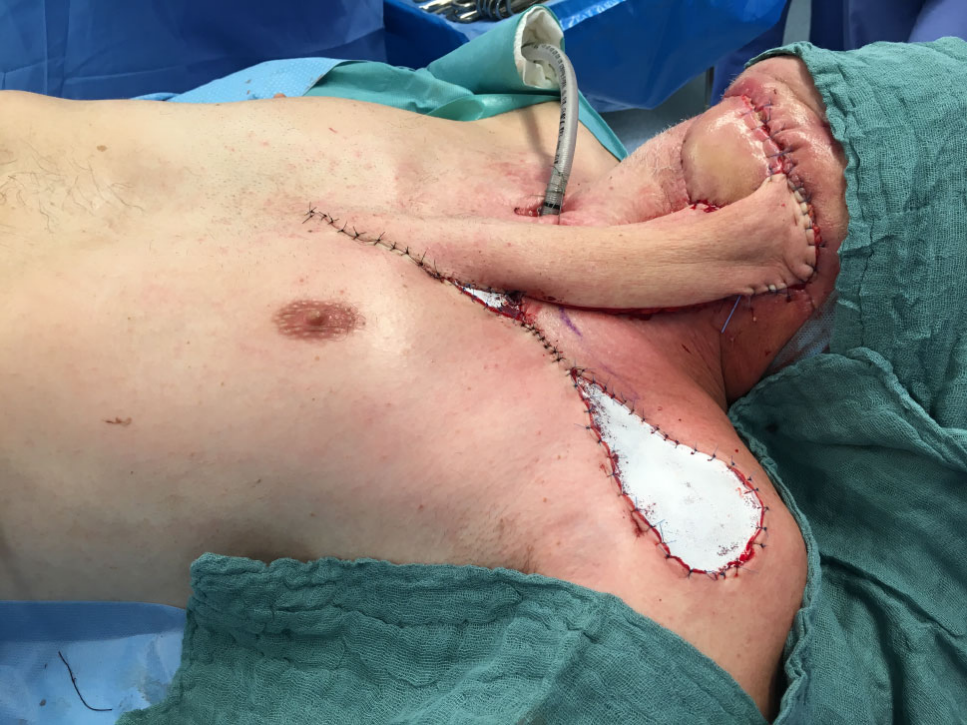
Back-up solution, surgery situation: A 57-years-old patient with an extraoral fistula after resection of a malignant tumor, radiation therapy, pathological fracture of the lower jaw due to an osteoradionecrosis and a free flap fibula reconstruction of the mandible, now treated with a deltopectoral flap for defect closure of a chronic extraoral fistula. Published with the patient's consent

**Fig. 4:**
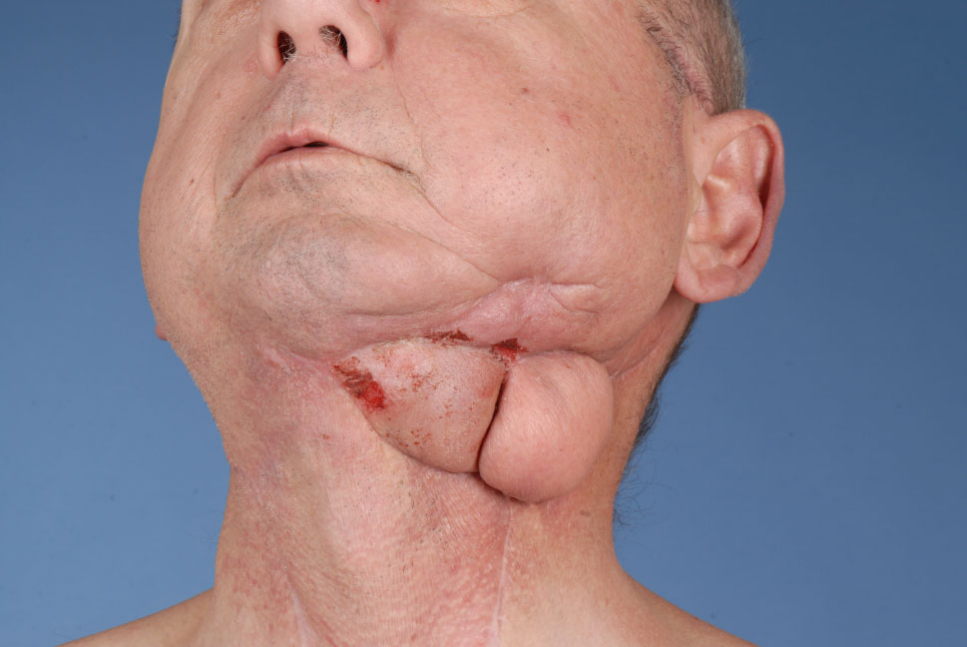
Back-up solution, follow-up situation after pedicle dissection of the deltopectoral flap with stable and completely healed wound. Published with the patient's consent

**Fig. 5:**
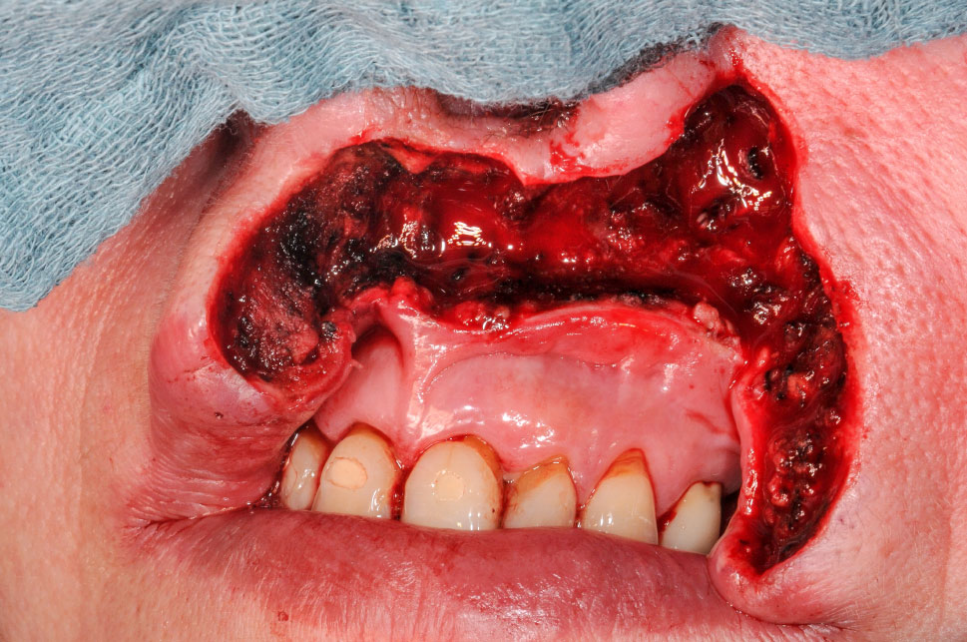
Esthetic solution: A 56-years-old patient after resection of a basal cell carcinoma of the left infranasal region and the upper lip. Published with the patient's consent

**Fig. 6:**
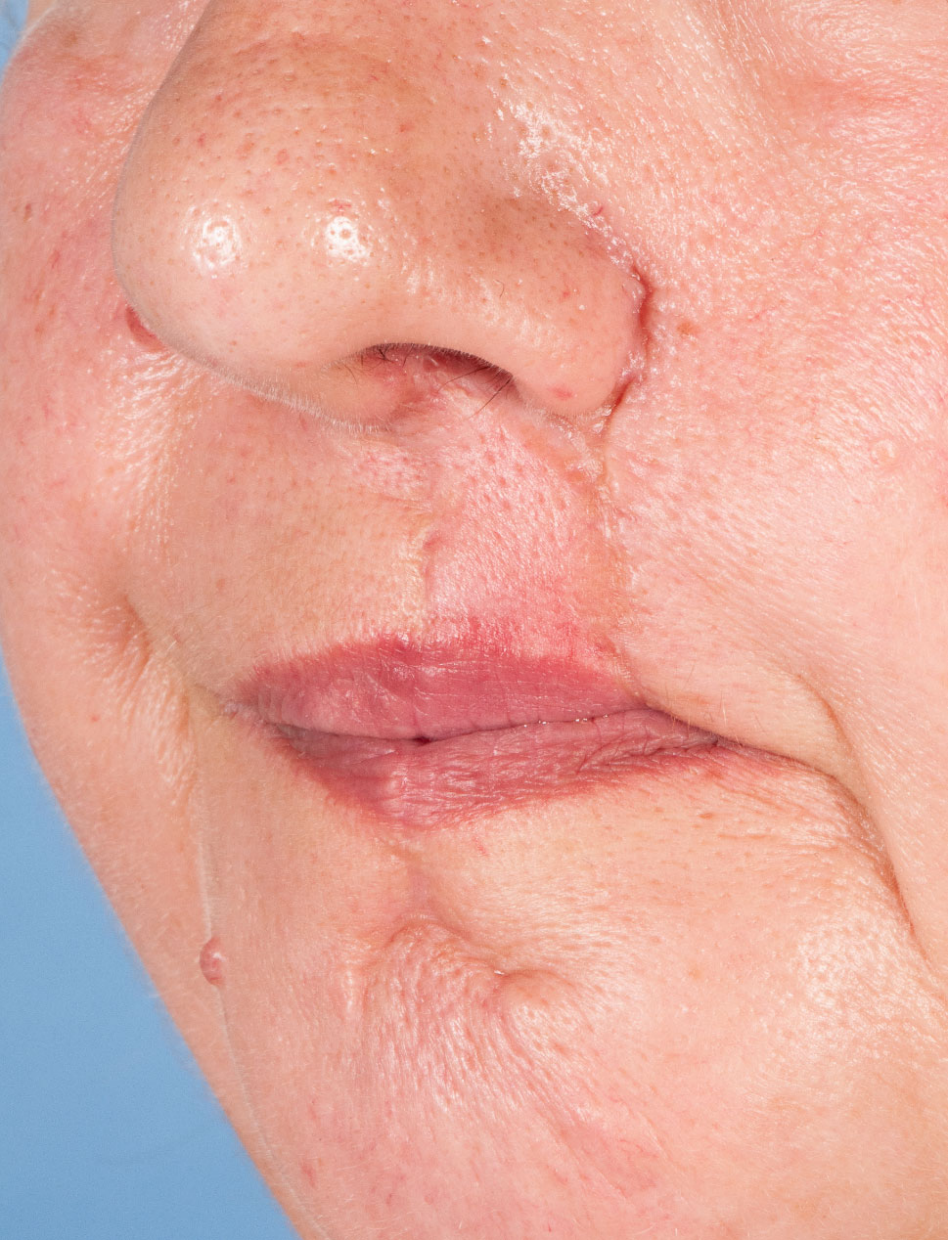
Esthetic solution: stable and completely healed wound situation of an Abbe plasty after dissection of the pedicle. Published with the patient's consent

Hence, most cases reviewed in this article (*n* = 25, 69.4%) revealed the presence of highly compromised, fibrotic, irradiated, scarred, fistulated, and even chronically infected tissue with a variety of harvested flaps (Figs. 3 and 4). In 11 (30.6%) cases, the flaps were selected as the best esthetic option for defect closure of the facial skin (Figs. 5 and 6). Hence, we clearly outline two major indications, deduced from a long reconstructive tradition, for two-stage pedicle flaps, namely the heavily pre-treated patient who is unsuitable for a long microsurgical procedure and the patient collective that is in need for an ideal reconstruction of the esthetic units of the facial skin were local pedicled flaps that show major advantages compared to microvascular procedures [2, 9]. Meaning that the texture and color of the facial skin cannot be imitated by any other harvestable tissue in the human body, local flaps serve as the ideal donor to preserve the facial profile [10]. Especially in nasal defects, the forehead flap is reliable for reconstructing full-thickness resections by preserving the functional and esthetic units of the face [11]. The same holds true for complex lip reconstruction [12]. The lip components are the oral mucosa, orbicularis oris muscle, and smaller mimic muscles, while the white role marks the border between the keratinized red part of the lip and the facial skin forming the cupid bow at the vermillocutaneous intersection. Mimicking these complex esthetic and functional interactions using microsurgical reconstruction is nearly impossible, leaving local flap procedures the only surgical option to achieve satisfactory results (Figs. 5 and 6) [13].

In terms of postsurgical complications, we noted that of the 10 (27.8%) flaps with compromised wound healing, majority occurred in the back-up pedicle flap group and only two events (1 [2.8%] venous congestion and 1 [2.8%] wound dehiscence) occurred in the group that received a pedicle flap for esthetic reasons. These results are in line with those of previous studies [5, 14] that postsurgical complications in pedicled flaps are significantly fewer than those in microvascular reconstruction in a vessel-depleted neck (34.5%) [15]. This could be attributed to the more predictable blood supply of local pedicle flaps that lie outside the field of radiation and favor fewer wound revisions. In our study, except in one patient who showed a persistent intraoral fistula, all wounds healed completely.

Current studies on donor site morbidity have shown that a pectoralis major flap does not cause greater donor site morbidity than a microvascular latissimus dorsi flap [16]. Interestingly, the radial forearm flap, which is the workhorse flap in reconstructive units for versatile applications due to its thin and adaptive lining, causes the highest donor site morbidity than any other pedicle or fasciocutaneous free flap [17, 18].

In spite of the several advantages in the use of pedicle flaps, some limitations must be considered. First, the disfiguring appearance of the pedicle for flap autonomation is a disadvantage as it precludes the patient from participation in a normal social life. While the optimal time of transplant autonomy from its blood supplying pedicle is not accurately determined in the current research, it could range from weeks to months [10, 19, 20]. These results are in line with our own experience that the time for pedicle dissection ranges from 14 to 106 days. Further research is needed to avoid a compromised facial appearance and to secure flap survival at the same time.

Furthermore, pedicle flaps do not allow full bone reconstruction of the maxilla or mandible. Correspondingly, if dental rehabilitation is planned, a harvested fibula flap, scapula flap, or deep circumflex iliac artery bone flap (DCIA) with microvascular anastomosis is the reconstruction of choice.

Finally, in terms of tumor surgery, the principles of mindful reconstruction should not be abandoned, and areas of lymphatic drainage from the pedicle flaps should be spared. If possible, the transposition of a cervical pedicle flap in a metastatic neck should be avoided to prevent the spread of tumor cells.

If these basic principles are considered, carefully selected patients will benefit from reconstruction by two-stage pedicle flaps in modern reconstructive maxillofacial surgery.

## Conclusion

The study outlines two indications for the use of two-stage pedicle flaps. First, as a back-up strategy in heavily pre-treated wound beds and second in an almost contrasting indication as the first-choice reconstructive option for the facial skin in esthetically demanding cases. Their timesaving and straightforward surgical approach, fewer postsurgical complications, and good long-term success rates have secured the two-stage pedicle flap a justified niche role in the age of microsurgical maxillofacial reconstruction.

## Supplementary Information

Below is the link to the electronic supplementary material.

Supplementary file1 (JPG 8964 KB)

## Data Availability

Additional chart data from all patients may be provided by contacting the corresponding author.
