# Supplementary figures and images for: Consistent Value of Two-Stage Pedicle Flaps in the Age of Microsurgical Maxillofacial Reconstruction
Source: J Maxillofac Oral Surg. 2021 Aug 24;22(Suppl 1):98–104. doi: 10.1007/s12663-021-01635-9 (PMC10082879; doi:10.1007/s12663-021-01635-9)

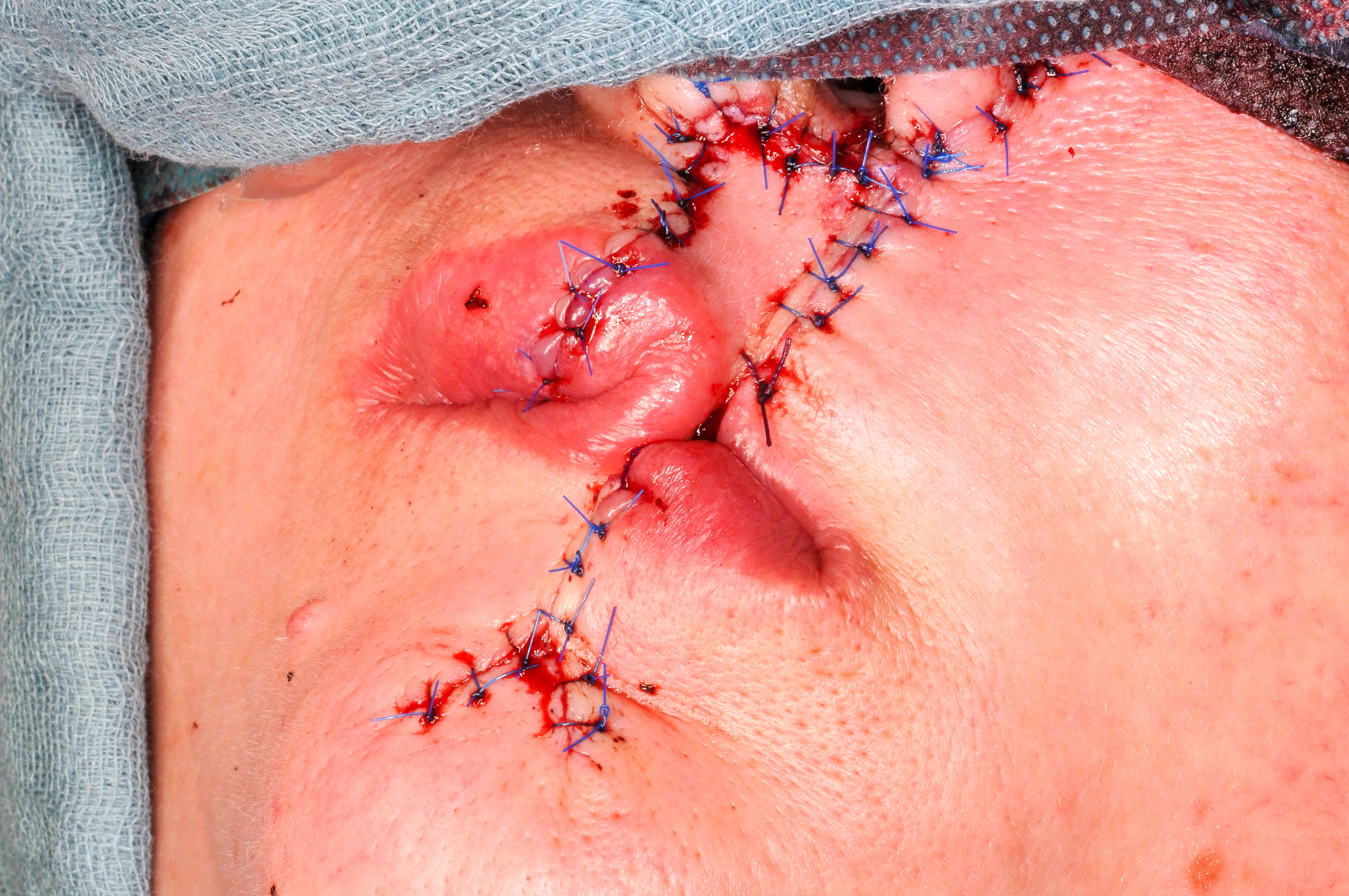

Supplement: Supplementary file 1 — Supplementary file1 (JPG 8964 KB) [file 12663_2021_1635_MOESM1_ESM.jpg]
